# Assessment of Dietary Selenium and Vitamin E on Laying Performance and Quality Parameters of Fresh and Stored Eggs in Japanese Quails

**DOI:** 10.3390/foods9091324

**Published:** 2020-09-20

**Authors:** Zabihollah Nemati, Hosain Ahmadian, Maghsoud Besharati, Steven Lesson, Kazem Alirezalu, Rubén Domínguez, José M. Lorenzo

**Affiliations:** 1 Department of Animal Science, University of Tabriz, Tabriz, East Azerbaijan 51666, Iran; znemati@tabrizu.ac.ir (Z.N.); ahmadianhosain6293@gmail.com (H.A.); m_besharati@hotmail.com (M.B.); 2 Department of Animal Sciences, University of Guelph, 50 Stone Road East Guelph, ON 53681, Canada; sleeson@uoguelph.ca; 3 Department of Food Science and Technology, Ahar Faculty of Agriculture and Natural Resources, University of Tabriz, Tabriz, East Azerbaijan 51666, Iran; kazem.alirezalu@tabrizu.ac.ir; 4 Centro Tecnológico de la Carne de Galicia, Rúa Galicia Nº 4, Parque Tecnológico de Galicia, San Cibrao das Viñas, 32900 Ourense, Spain; jmlorenzo@ceteca.net; 5 Área de Tecnología de los Alimentos, Facultad de Ciencias de Ourense, Universidad de Vigo, 32004 Ourense, Spain

**Keywords:** antioxidant, blood biochemical parameters, production performance

## Abstract

The effect of dietary supplementation with VE and Se on the laying productive performance, immunity, and the quality parameters of fresh and stored eggs was assessed. For this study, five treatments, namely control (basal diet), control plus 30 mg of VE and 0.4 mg kg^−1^ sodium selenite (VE30SS), control plus 30 mg of VE and 0.4 mg kg^−1^ of Sel-Plex^®^ (VE30SP), control plus 120 mg VE and 0.4 mg kg^−1^ Sodium selenite (VE120SS), and control plus 120 mg VE and 0.4 mg kg^−1^ Sel-Plex (VE120SP), were examined. There was no huge impact of VE and Se on feed consumption, FCR and egg yield rate. Quality parameters of fresh egg including egg surface area, eggshell thickness, yolk selenium concentration, albumen height, and Haugh unit were significantly increased following VE and Se supplementation (*p* < 0.05). For stored eggs, VE and Se significantly increased egg yolk color intensity (*p* < 0.05). Regardless of storage temperature, eggs from birds fed with VE and Se had less weight loss during 30 days of storage. Albumen height was significantly higher in VE and Se fed birds in eggs stored at 5 °C for 15 and 30 days. The combination of Sel-Plex with either levels of VE had significantly higher blood total antioxidant capacity. Dietary VE and Selenium, notably Sel-Plex, improved the antioxidant potential of blood and egg quality of laying quails.

## 1. Introduction

Under normal circumstances, excessive free radicals are scavenged by body enzymatic and non-enzymatic antioxidants, but oxidative stress can occur when the amount of oxidant production surpasses the capacity of the body’s antioxidant system [1,2]. Different aspects of avian productive and reproductive performances, such as egg production rate and egg quality traits, as well as fertility and hatchability may be adversely influenced by oxidative stress.

Selenium (Se) is the key constituent of several seleno-proteins and enzymes involved in antioxidant system, DNA repair, fertility, and productive traits [3]. There are both organic and inorganic compounds of Se in nature. Selenomethionine is the main form of organic selenium, whereas sodium selenite (Na_2_SeO_3_) is an inorganic source of selenium. Regardless of the fact that Se requirement of birds can usually be met by corn and soybean meal diets, dietary sodium selenite up to 0.5 mg kg^−1^ (maximum level) is usually recommended to improve avian health and productive performance [4]. Sel-Plex (SP) is a commercial name for organic selenium that is characterized by yeast *Saccharomyces cerevisiae* growth in Selenium-rich culture medium [5]. Birds cannot synthesize selenomethionine by an effective mechanism, and therefore rely on grass and forage crops that transform selenium primarily into selenomethionine and incorporate this into nitrogen components instead of methionine [5]. Unlike inorganic selenium, with intestinal passive absorption, selenomethionine is actively absorbed through the mechanism used for the absorption of methionine. Higher duodenal absorption and deposition in the liver make organic selenium an interesting Se source in laying hens to meet their requirement without sacrificing performance [6]. In addition, lower excretion rate and the ability to incorporate into the structure of proteins provides the body with a natural and reversible source of selenium storage [7,8]. On the other hand, organic selenium seems more effective than the inorganic form in improving egg quality [9] and bird immunity [10]. Mobaraki and Shahryar [11] showed that dietary selenium (0.4 mg/kg) and vitamin E (160 mg/kg) had no significant effect on feed intake, shell thickness, the weight of egg whites and the percentage of egg hatchability stored for seven days. However, they reported that the treatments of the study did not have significant impacts on other features, i.e., egg production percentage, feed conversion ratio, eggshell percentage, yolk weight percentage, international quality unit (IQU), Haugh unit, and percentage egg hatchability in 10 days storage. Previous studies have reported Selenium supplementation of Japanese quail diets improved the egg shelf life during storage period by improving HU and decreasing yolk fat peroxidation [12].

Vitamin E (VE) is the main antioxidant in egg yolk lipid terminating the lipid peroxidation chain by reacting with lipid peroxides and making them stable [2]. The use of excess dietary vitamin E in poultry’ feed has been shown to increase the growth performance, the meat nutritional value in terms of the fatty acid composition, and the shelf life [13]. Łukaszewicz et al. [14] determined the effect of feed supplementation with organic selenium and vitamin E on chemical composition and sensory characteristics of Japanese quail (*coturnix japonica*) eggs and reported that feeding Japanese quail with selenium and vitamin E reduces the concentration of fat in eggs and the concentration of cholesterol in yolk, while increasing the levels of selenium, protein, and antioxidant properties. In addition, feeding 60 IU kg^−1^ of VE results in improved of feed intake, laying percentage and egg quality traits including Haugh unit, egg weight and vitelline membrane strength [15]. There is undoubtedly a strong relationship between Se and VE in terms of protecting cells against free radical damage [3]. Moreover, their metabolisms are closely interrelated since the absorption of vitamin E is impaired in birds during Se deficiency [16]. Dietary Se also increases VE content of plasma, egg yolk and meat of chickens [17]. Therefore, it is not surprising that researchers have focused on applying the Se and VE together in their studies.

The beneficial effects of Se and VE on the rate of egg production and fresh and stored egg quality traits have been extensively studied in laying hens [18,19] but information regarding the potential benefits of these antioxidants on the productive performance of Japanese quails is limited. Thus, the current experiment was conducted to survey the effectiveness of different VE (30 or 120 mg kg^−1^) and selenium sources (organic or inorganic) on the fresh or stored egg quality parameters and productive performance in Japanese quails.

## 2. Materials and Methods

The experimental protocols were recorded according to the regulations for the use of experimental animals and approved by the Ethical Committee for Use of Laboratory Animals of University of Tabriz (Approved number: IR.TABRIZU.REC.1399.035).

### 2.1. Chemical Agents and Experimental Design

All chemicals with the purity of >99% (analytical grade) were prepared from Merck (Darmstadt, Germany). Selenium and vitamin E were purchased from Sigma Aldrich (SEM-AZ, Baku, Azerbaijan).

A total of 300 female 12-week-old Japanese quails with similar body weight and production rate were randomly distributed into five groups with four replicates of fifteen birds in each. Birds were group caged in a well-ventilated room under 8:16 dark: light lighting regime. Animals received water and feed ad libitum throughout the study. Treatment groups consisted of basal diet (control), basal diet addition 30 mg of VE and 0.4 mg kg^−1^ sodium selenite (VE30SS), basal diet addition 30 mg of VE and 0.4 mg kg^−1^ of Sel-Plex (VE30SP), basal diet addition 120 mg of VE and 0.4 mg kg^−1^ of Sodium selenite (VE120SS) and basal diet addition 120 mg of VE and 0.4 mg kg^−1^ of Sel-Plex (VE120SP). Sodium selenite (containing 46% Se), Sel-Plex (containing 0.1% Se), and vitamin E (α-tocopherol acetate) were included in the feed via premix. The basal diet (Table 1) was formulated according to recommendation of NRC [20].

### 2.2. Determination of Productive Traits and Egg Quality

Egg production rate, egg weight and feed consumption were recorded week by week, whereas feed conversion rate and egg mass were determined at the end of experimental period. For an accurate evaluate of the interior and exterior fresh egg quality traits, six eggs were randomly gathered from each replicate treatment at the end of weeks 2, 4, 6, and 8 (a sum of 24 eggs of each treatment). Stored egg parameters were evaluated on eggs (*n* = 24 eggs of each treatment) collected at the end of trial (week 8) and were then stored for 15 or 30 days at 5 or 22 °C. Measurements were made according to the equations presented in the footnote of Table 2. Using 0.01 mm digital caliper, egg width and height and subsequently shape index were determined. Eggs from each treatment were weighed one by one and all of them broken open onto a flat plate.

Thick albumen height was measured using a 0.01 mm digital caliper. Haugh unit for each egg was calculated using data of egg weight and albumen height. In order to determine eggshell percentage, eggshells after washing with warm water were dried at 100 °C for 4 h and weighed with a 0.01 g in an electronic digital balance. Shell thickness measuring gauge was used to estimate shell thickness at 3 different unique focuses (blunt, equator and sharp end).

Egg yolk and egg albumen pH were determined on fresh and stored eggs. Briefly, egg content was separated to yolk and albumen using an egg separator. Individual egg yolk color was measured by using Roche color fan. Then, they were poured into different glass beakers, homogenized with a stirring bar, and finally their pH value was measured with a standard pH-meter. At the end of 8-week trial period, the yolk of all eggs in each experimental replicate were mixed together and egg yolk selenium and malondialdehyde concentrations assayed on fresh and stored eggs, respectively. To determine Se concentration, briefly, yolk samples were processed in a blend of H_2_O_2_ and HNO_3_ in Teflon High-pressure vessels in an MDS-2000 microwave oven. After mineralization, Se concentration was quantified by electro thermal atomizer in a graphite cuvette, employing the solar M6 atomic absorption spectrometer. The method was validated by analysis of the Se content of whole egg powder (NIST RM 8415) [21]. The thiobarbituric acid reactive substances (TBARS) assay with small modification was used to measure lipid oxidation [22]. Briefly, 2 grams of homogenous yolk was mixed with 5 mL of 20% trichloroacetic acid (TCA) and 4 mL distilled water, and homogenized in a Waring Blender for 30 s at high speed. The homogenate was then centrifuged (1000 g for 20 min) and the resulting supernatant was filtered with Whatman Filter Paper (grade no. 1). Two mL of filtrated was mixed with 2 mL of thiobarbituric acid (TBA, 0.02 M) in a test tube and heated in boiling water for 20 min. After cooling, the absorbance of the resulting solution was record with a spectrophotometer at 532 nm (Jenway 6405, Staffordshire, UK).

### 2.3. Blood Biochemical Parameters

At the end week of experimental period (8th week), three birds were selected from each treatment groups and blood samples were collected from their neck vein. After collection, samples were carefully transferred to tubes containing K2EDTA as an anticoagulant. They were then centrifuged (1,500 g, 10 min, 15 °C) and the plasma samples after separation were stored at −20 °C until analyzed for total antioxidant capacity (TAC), cholesterol and triglyceride concentrations. TAC of blood samples was assessed by using ferric reducing antioxidant power bioassay [23]. In this method, antioxidants present in the samples are used as reductants to reduce Fe^3+^ to Fe^2+^. Therefore, TAC was quantified by the response of phenanthroline and Fe^2+^ utilizing a spectrophotometer reset at 520 nm. A unit of TAC is characterized as the amount of antioxidants expected to increase absorbance value at 0.01 in 1 mL of blood sample at 37 °C. Blood cholesterol and triglyceride were measured by spectrophotometric instrument (JENWAY-6405), employing commercially available kits (ZiestChemie Diagnostic, Tehran, Iran).

### 2.4. Statistical Analysis

The experimental data including blood biochemical parameters of Japanese quails, egg yolk selenium concentration and egg quality traits of stored eggs were analyzed statistically based on a completely randomized design with ANOVA using the GLM procedure of SAS (2009). The differences among the groups were determined using Duncan’s multiple range tests. Significance was accepted at *p* < 0.05. Data for productive performance and fresh egg quality traits were analyzed using repeated measures analysis and the Proc Mixed procedure of SAS with 5 experimental diets, and 4 rearing phase as fixed effects. Mean differences were compared using Tukey’s tests at the *p* < 0.05 level.

## 3. Results

### 3.1. Production Performance

The effect of VE and Se supplementation diet on quail’s performance are present in Table 2. The egg production, feed consumption and feed conversion ratio of birds fed dietary supplementation of VE and Se for the eight-week experimental period did not differ significantly (*p*> 0.05).

**Figure 1:**
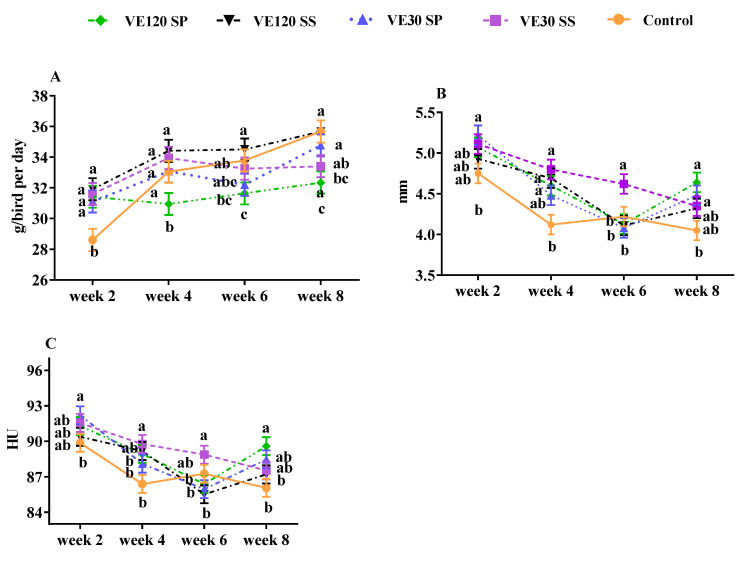
Variation of feed intake (**A**), albumen height (**B**) and Haugh unit of fresh eggs (**C**) from Japanese quails fed different levels of vitamin E (VE, 30 or 120 mg kg^−1^) and Se sources (SS, sodium selenite; SP, Sel-Plex) over a period of eight weeks. Values (means ±SEM) within the same week with uncommon letters are significantly (*p* < 0.05) different.

Variation of food consumption during the eight weeks has been presented in Figure 1A. Apart from main effect of treatments, food consumption increased in all birds during the current study (Figure 1A). Egg weight showed a tendency to improve (*p* = 0.05), but egg mass was not affected by Se sources and various levels of vitamin E, as shown in Table 2 (*p* > 0.05).

### 3.2. Quality Traits of Fresh and Stored Eggs

Some egg quality traits including Haugh unit, albumen height, eggshell thickness and egg surface area were significantly affected by VE or Se supplementation (*p* < 0.05), but other egg traits including albumen and yolk percentage, egg specific quality, yolk albumen ratio, eggshell percentage and strength and egg shape index were not influenced by VE or Se supplementation as shown in Table 2 (*p* > 0.05). Albumen height and Haugh unit showed a downward trend during eight experimental periods (Figure 1B,C).

**Figure 2:**
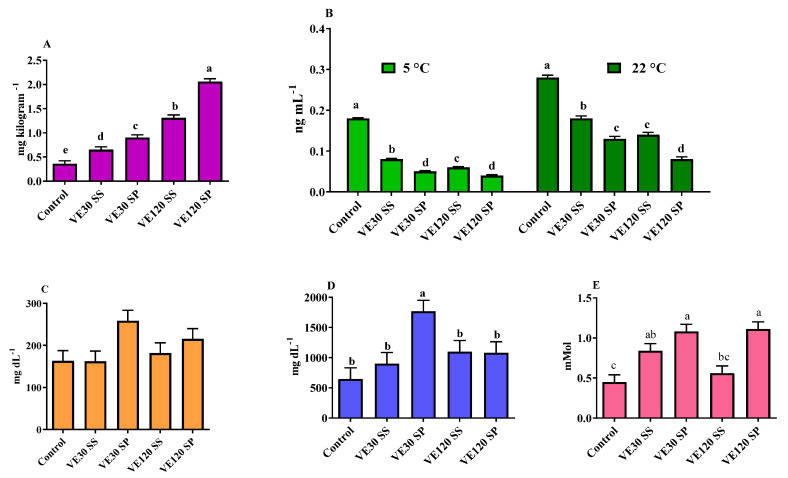
(**A**) egg yolk selenium concentration, (**B**) egg yolk MDA concentration after being stored for 30 days at 5 and 22 °C, (**C**) blood cholesterol concentration, (**D**) blood triacylglycerol concentration and (**E**) blood total antioxidant capacity of Japanese quails fed different levels of vitamin E (VE, 30 or 120 mg kg^−1^) and Se sources (SS, sodium selenite; SP, Sel-Plex). Values (means ± SEM) with uncommon letters are significantly (*p* < 0.05) different in each graph.

Organic and inorganic form of dietary Se dose-responsively (*p* < 0.05) improved Se content in egg yolks, with a more effective on Se deposition according to Sel-Plex (Figure 2A). In birds fed with Sel-Plex, the Se concentration of egg yolks were 0.90 and 2.06 mg kg^−1^, for VE30SP and VE120SP groups, respectively. Correspondingly, egg yolk from birds fed with sodium selenite had 0.65 and 1.31 mg kg^−1^ Se, for VE30SS and VE120SS groups, respectively.

Egg yolk MDA concentration, indicative of fatty acid oxidative stability, increased during the 30 days of storage at either 5 or 22 °C with the highest value observed for those stored at ambient temperature (Figure 2B). Irrespective of treatment levels, beneficial effects were observed in all birds receiving VE and Se sources compared to control birds. Moreover, the combination VE and Sel-Plex was more effective to delay the lipid peroxidation compared to the combination of VE and sodium selenite. However, it was only VE120SP group which recorded the lowest significant yolk MDA concentration among treated groups (*p* < 0.05, Figure 2B).

In this study, without regard to storage room temperatures, the egg weight loss in percentage was significantly affected at both storage times with a more prominent effect in treatments with 120 mg of VE (Table 3).

### 3.3. Blood Parameters

Dietary VE and Se did not influence blood cholesterol concentration (Figure 2C). The concentration of plasma triacylglycerol (Figure 2D) was significantly increased only in VE30SP group (1767) compared with control (647.44) and birds fed with other combinations of VE and Se (902, 1101 and 1080, for VE30SS, VE120SS and VE120SP, respectively). The plasma total antioxidant capacity value (*p* < 0.05) was highest in birds fed with the combination of VE (30 or 120 mg) and Sel-Plex compared to control group (Figure 2E).

## 4. Discussion

The results of this study clearly indicated that dietary supplementation of VE and Se had no critical effect on feed consumption, FCR and egg production of Japanese quails (Table 2). Consistent with our results, dietary VE levels (30 or 120 mg/kg) with either organic or inorganic selenium source did not influence egg production in laying hen [19]. In addition, egg production rate was not influenced by increasing organic or inorganic level of selenium [24]. Likewise, the average daily gain, average daily feed intake, and feed conversion ratio of broiler chicks were not affected by dietary supplementation of various selenium sources [6]. However, apart from main effect of treatments, food consumption increased in all birds during the current study (Figure 1A). This effect is consistent with data reported by Skrobanek et al. [24] who observed a direct relationship between feed intake and aging. In terms of egg production, our results agreed with those observed by Mobaraki and Shahryar [11] who noticed that similar levels of VE and Se sources did not significantly affect laying rate during a period of 25 weeks. Other studies also noted that the increasing dietary vitamin E levels also have no significant effect on laying percentage [25,26]. By contrast, a significant increase in feed intake and percentage of egg production was found in hens raised at both natural (21 °C) and higher (34 °C) ambient temperatures [15].

In the current study, egg weight showed a tendency to improve (*p* = 0.05) by various levels of vitamin E and selenium sources (Table 2). According to Reis et al. [27] Se (from 0.15 to 0.30 mg kg^−1^) had no significant effect on egg weight of broiler breeders. On the contrary, Urso et al. [19] and Mobaraki and Shahryar [11] reported a higher egg weight in hens fed similar amount of VE and organic selenium. Vitamin E through its ability to protect liver and other organs integrity may preventing oxidative damage, thereby induce yolk precursor protein release which necessary to egg formation [28]. Otherwise selenium is essential for metabolism and action of thyroid hormones in body [29] that could be affect egg weight.

The most common egg quality characteristics are egg shape index, breaking strength, shell thickness, specific gravity, albumen height and weight, pH of albumen and yolk, Haugh unit and oxidative status of yolk lipids [30,31]. Despite having a significant effect on Haugh unit, albumen height, eggshell thickness, and egg surface area (*p* < 0.05), other egg traits were not influenced (*p* > 0.05) by VE or Se supplementation (Table 2). As Payne et al. [32] and Arpasova et al. [9] previously pointed out, hens consumed increasing levels of organic Se had significantly higher Haugh unit. Furthermore, according to Maysa et al. [33] Haugh unit, egg yolk index, and shell thickness were significantly higher in Sel-Plex fed hens.

Reports of the effect of VE supplementation on egg internal quality are contradictory and inconclusive. In one study, supplementation of vitamin E had no significant effect on albumen pH and eggshell thickness [25]. However, in another one, egg specific gravity, eggshell weight, and eggshell strength of laying hens were significantly increased after being fed with a diet comprised 250 mg/kg of VE [34].

Regardless of source, dietary Se supplementation was proposed to deposit more selenium in egg yolk than albumen [35]. In our study, both forms of dietary Se dose-responsively (*p* < 0.05) enhanced Se content in egg yolks, with a high prominence effect seen in Sel-Plex (Figure 2A). Having more efficient deposition in the organic form, selenium content of albumen-yolk and diet were directly related [3,35]. More importantly, organic selenium was reported to be much more effective than the inorganic form in maintaining egg storage quality, and most notably eggshell strength [36]. Not only is eggshell an extra Se source for developing embryos, but it also plays a physiological role in the physical protection to the embryo, by means gas exchange and ionic trade and water regulation [37]. It seems that improving fresh egg quality traits in the current study at least in part related to the Se deposition in quail’s eggs since egg quality indicators has been proposed to directly correlate with organic selenium concentration in egg [38]. Albumen height and Haugh unit showed a downward trend during our experiment (Figure 1B,C). Deteriorating effects of aging on egg quality has been noted in earlier studies. For instance, the albumen index, Haugh unit, and yolk index of eggs from 21-week-old quails were significantly lower compared to younger (12-week-old) birds [39].

Egg storage is usually inevitable prior to incubation or consumption. However, this may adversely affect egg quality, most probably through decline of carbon dioxide and raising the pH of albumen and yolk [40]. Phosvitin is a phosphoglycoprotein in egg yolk inhibiting metal-catalyzed phospholipid oxidation. It was shown that the pH-induced changes in phosvitin conformation, impairs its metal binding activity and consequently its antioxidant activity [41]. This may result in altering the oxidative stability of egg yolk phospholipids. In addition, having approximately 60% unsaturated fatty acids make egg yolk phospholipids very susceptible to lipid peroxidation during storage [42]. Seen in this light, measuring malondialdehyde (MDA) levels offers a convenient method of determining lipid peroxidation in egg yolk. In our study, irrespective of treatment levels, beneficial effects were observed in all birds receiving VE and Se sources compared to control birds. The lower values of MDA in all birds receiving VE and Se sources are probably due to selenium accumulation in egg yolk. Also, the lowest significant yolk MDA concentration in VE120SP group (*p* < 0.05, Figure 2B) compared to other treated groups may be due to the better accumulation of Sel-Plex in egg yolk. In addition, enhancing alpha-tocopherol contents of egg yolk by dietary Se supplementation might be involved in antioxidant potential of egg yolk [16]. The protective effect of VE and Se on lipid peroxidation might be as a result of their antioxidant properties in GSH-Px activity. Being involved in the detoxification of pro-oxidants, the reduced form of GSH-Px reduces hydrogen peroxide and lipid hydroperoxide at the level of the cytosol and mitochondrial matrix [43]. In conformity with our information, Mohti-Asli et al. [18] found that the increasing trend of MDA concentration in eggs stored in room temperature and incubator can be mitigated through maternal dietary vitamin E and selenium.

It appeared that the storage time and temperature are the two most crucial factors influencing egg quality during storage. Egg weight loss through evaporation is affected by the relative humidity and temperature during long-term storage conditions. In the present study, regardless of storage temperatures, the egg weight loss was significantly affected at both storage times with more prominence effect seen in treatments with 120 mg of VE (Table 3). Apart from environmental factors, some quality traits including shell porosity and shell thickness involve in egg weight loss [44]. Thus, the probable reason for minimum egg weight loss in birds fed selenium and VE in the current study might be related to their better eggshell quality such as higher shell thickness (Table 3). In agreement with our findings, egg weight reduction was shown to be tempered following dietary supplementation of Se in laying hens [3].

In the current experiment, albumen height and consequently Haugh unit were significantly improved in VE and Se supplemented groups during storage at 5 °C (Table 3). It seems that maintaining viscosity of albumen is the probable mechanism involved since viscosity and Haugh unit reduction were proposed with the progress in the storage period [16]. On the other hand, yolk pH was significantly lower after 15 or 30 days of storage at 5 °C compared to respective control groups (Table 3). As previously mentioned, lower egg yolk pH in this study may be as a result of higher antioxidant potential and consequently lower oxidative stress in stored eggs.

The antioxidant properties of VE and Se may also have influenced the color intensity of egg yolk. The responsibility of egg yolk color is oxycarotenoid pigments, which are derived from diet. These pigments are lipid soluble and their intestinal absorption associates with the absorption of lipids. When oxidized, oxycarotenoids lose their pigmenting properties. Therefore, the antioxidants present in the body or diet help maintain the stability of oxycarotenoids. In our study, the improvement of yolk color in stored eggs likely result from the antioxidant potential of dietary VE and selenium content.

In our study, dietary addition of VE and Se did not influence blood cholesterol concentration (Figure 2C). Mohiti-Asli et al. [18] also observed no significant effect of VE and Se supplementation on blood cholesterol concentration in laying hens. However, in another study, feeding with VE and Se decreased blood cholesterol concentration in heat-stressed Japanese quails [45]. The concentration of plasma triacylglycerol (Figure 2D) was significantly enhanced only in VE30SP group compared to control treatment and birds fed with other combinations of VE and Se. Thyroid hormone at physiological level is one of most potent agents known to reduce serum LDL concentration. Being involved in hepatic conversation of thyroxine to 3, 3, 5-triiodothyronine, selenium supplementation may lower plasma cholesterol concentration through its key role in thyroid hormone metabolism [46]. TAC of plasma fluctuated and the highest significant figures (*p* < 0.05) were recorded in birds fed with the combination of VE (30 or 120 mg) and Sel-Plex compared to control group (Figure 2E). Similar with our results, increasing dietary Se levels increased the TAC content of laying hen [46]. Selenium is an indispensable part of glutathione peroxidase enzyme and its combination with VE may protect the body against cellular lipid peroxidation [46,47].

## 5. Conclusions

In conclusion, Japanese quail dietary supplementation of different VE levels (30 or 120 mg kg^−1^) and organic or inorganic Se sources enhanced fresh and stored egg quality and the nutritional value of egg in terms of selenium, vitamin E, and egg yolk MDA concentration. By taking all data into consideration, 120 mg kg^−1^ of VE along with Sel-Plex provide birds with more successful results for assessed parameters. Furthermore, these results provide a commercial benefit during egg shipment and storage by the possibility of improving the maintenance of egg quality, and thereby have important practical significance for human as Se-enriched food.

## Figures and Tables

**Table 1:** Ingredient and composition of the basal diet.

Item	Value (%)	Composition	
Corn	58.9	ME (kcal/kg)	2900
Soybean meal	30	CP (%)	18
Soybean oil	3.2	Ca (%)	2.5
Dicalcium phosphate	1.2	Available P (%)	0.6
Calcium carbonate	5.7	Methionine (%)	2
Methionine	0.20	Lysine (%)	1.08
Sodium chloride	0.2	DCAB	220
Bicarbonate	0.1		
Vitamin premix ^1^	0.25		
Trace mineral premix ^2^	0.25		

^1^ Supplied per kg premix: vitamin B1: 720 mg, vitamin B2: 2640 mg, pantothenic acid: 4000 mg, nicotinic acid: 12,000, vitamin B6 1200 mg, folic acid: 400 mg, biotin; 40 mg, vitamin K3 800 mg, vitamin E; 7200 IU, chloin chloride: 100,000, antioxidant, 40,000 mg, vitamin A; 3,600,000 IU, vitamin D3; 800,000 IU and vitamin B12 6 mg; ^2^ Supplied per kg premix: FeSo4, 50 g; MnSo4, 40 g; CuSo4, 10 g; I, 400 mg and Se, 80 mg.

**Table 2:** Productive performance and fresh egg quality traits of Japanese quails fed different levels of VE and Selenium sources.

Traits	Treatments ^1^					SEM	*p* Value		
	Control	VE30SS	VE30SP	VE120SS	VE120SP		Treat	Time	Treat × Time
Feed intake ^2^	32.78	33.05	32.79	34.13	31.59	0.53	0.06	<0.01	<0.01
Feed conversion ratio ^3^	3.08	2.98	2.90	2.95	2.99	0.11	0.86	<0.01	0.07
Egg production ^4^	84.98	84.65	88.19	87.70	82.18	2.61	0.50	0.01	0.07
Egg weight ^5^	12.62	13.19	12.82	13.21	12.85	0.14	0.05	0.06	0.57
Egg mass ^6^	10.73	11.16	11.31	11.59	10.56	0.39	0.37	<0.01	0.10
**Fresh Egg quality traits**									
Egg specific gravity ^7^	1.07	1.07	1.07	1.07	1.07	0.00	0.95	0.49	0.66
Albumen height (mm)	4.28 ^b^	4.72 ^a^	4.56 ^a^	4.51 ^a^	4.61^a^	0.07	0.01	<0.01	0.01
Albumen percentage ^8^	60.75	60.55	60.24	60.28	60.43	0.22	0.52	<0.01	0.58
Yolk percentage ^9^	31.59	31.79	32.01	32.04	31.83	0.21	0.58	0.01	0.49
Yolk Albumen ratio ^10^	0.52	0.52	0.53	0.51	0.52	0.01	0.69	0.04	0.71
Haugh unit ^11^	87.39 ^b^	89.44 ^a^	88.68 ^a,b^	88.07 ^a,b^	89.05 ^a^	0.46	0.04	<0.01	<0.01
Eggshell percentage ^12^	7.64	7.65	7.74	7.67	7.73	0.10	0.95	0.49	0.66
Eggshell thickness (mm)	0.20 ^b^	0.21 ^a^	0.21 ^a^	0.21 ^a^	0.21 ^a^	0.002	0.02	0.02	0.41
Eggshell strength (hg/cm^2^)	19.22	19.24	19.46	19.28	19.43	0.27	0.95	0.49	0.66
Egg surface area ^13^	51.07 ^c^	53.50 ^a,b^	52.48 ^b,c^	54.55 ^a^	52.01 ^b,c^	0.63	0.01	0.01	0.19
Egg shape index ^14^	78.43	73.10	79.18	78.34	78.22	2.42	0.42	0.43	0.53

^1^ Treatments were including different levels of vitamin E (VE, 30 or 120 mg kg^−1^) and Se sources (SS, sodium selenite; SP, Sel-Plex); ^2^ gram/bird per day; ^3^ feed conversion ratio = feed intake/egg mass; ^4^ Hen-day egg production = (total number of eggs/number of live layers) × 100; ^5^ average egg weight = total weight of collected eggs/total number of collected eggs; ^6^ Egg mass = [egg production (%) × average egg weight (g)] × 100; ^7^ Egg specific gravity = egg weight/[0.9680 × (egg weight – Shell weight) + (0.4921 × shell weight)]; ^8^ Albumen percentage = (albumen weight/egg weight) ×100; ^9^ Yolk percentage = (yolk weight/egg weight) × 100; ^10^ Yolk Albumen ratio = yolk weight/albumen weight; ^11^ Haugh unit = 100 log [albumen height (mm)—1.7 weight of the egg (g)0.37+ 7.57]; ^12^ Eggshell percentage = (dried shell weight/egg weight) × 100; ^13^ Egg surface area (mm) = 3.9782 × egg weight (g); ^14^ Egg shape index = (egg width/egg length) × 100. Values (means) within a row with uncommon letters are significantly (*p* < 0.05) different.

**Table 3:** Quality traits of stored eggs from Japanese quails fed different levels of vitamin E and selenium sources.

Temperature (°C)	Time (day)	Treatments ^1^	Traits					
			Egg Weight Loss (%)^2^	Albumen Height (mm)	Yolk Colour	Yolk pH	Albumen pH	Haugh Unit
5	15	Control	0.44	2.80 ^b^	4.45 ^c^	7.02 ^a^	8.68	78.52 ^b^
		VE30SS	0.37	3.24 ^a^	4.94 ^b,c^	6.48 ^b^	8.85	81.18 ^a^
		VE30SP	0.67	3.24 ^a^	5.41 ^b^	6.23 ^b^	8.56	81.26 ^a^
		VE120SS	0.33	3.35 ^a^	5.32 ^b^	6.29 ^b^	8.88	81.35 ^a^
		VE120SP	0.40	3.15 ^a^	6.18 ^a^	6.34 ^b^	8.86	80.48 ^a^
		SEM	0.14	0.08	0.22	0.16	0.13	0.58
		*p value*	0.49	<0.01	<0.01	0.02	0.37	0.01
	30	Control	1.94 ^a^	2.14 ^b^	4.50 ^d^	6.75 ^a^	8.76	74.99 ^b^
		VE30SS	1.48 ^b^	3.23 ^a^	4.90 ^c^	6.31 ^b^	8.75	80.65 ^a^
		VE30SP	2.07 ^a^	3.16 ^a^	5.08 ^b^	6.46 ^b^	8.71	80.61 ^a^
		VE120SS	1.43 ^b^	3.54 ^a^	5.05 ^b,c^	6.44 ^b^	8.65	82.96 ^a^
		VE120SP	1.52 ^b^	3.54 ^a^	6.19 ^a^	6.42 ^b^	8.74	83.26 ^a^
		SEM	0.13	0.15	0.14	0.08	0.04	1.07
		*p value*	<0.01	<0.01	<0.01	0.03	0.57	<0.01
22	15	Control	2.94	2.90	4.30 ^c^	6.33	9.26	78.69
		VE30SS	3.46	3.13	5.81 ^b^	6.31	9.24	80.04
		VE30SP	3.28	2.97	6.16 ^a,b^	6.32	9.27	79.66
		VE120SS	3.00	2.99	6.27 ^a,b^	6.33	8.50	79.19
		VE120SP	3.47	2.93	6.50 ^a^	6.34	9.27	79.46
		SEM	0.25	0.08	0.21	0.04	0.33	0.62
		*p value*	0.55	0.42	<0.01	0.98	0.41	0.63
	30	Control	7.77 ^A^	2.56	4.33 ^D^	6.67 ^A^	9.27	77.15 ^b^
		VE30SS	7.37 ^A,B,C^	2.56	5.11 ^B,C^	6.65 ^A,B^	9.29	76.81 ^b^
		VE30SP	7.53 ^A,B^	2.71	5.69 ^A,B^	6.48 ^B,C^	9.29	78.14 ^a,b^
		VE120SS	6.99 ^C^	2.59	5.03 ^C^	6.49 ^B,C^	9.29	76.77 ^b^
		VE120SP	7.14 ^B,C^	2.72	6.24 ^A^	6.47 ^C^	9.27	78.63 ^a^
		SEM	0.16	0.07	0.21	0.05	0.01	0.46
		*p value*	0.02	0.36	<0.01	0.04	0.47	0.04

^1^ Treatments were including different levels of vitamin E (VE, 30 or 120 mg kg^−1^) and Se sources (SS, sodium selenite; SP, Sel-Plex); ^2^ Egg weight loss (%) = [(initial egg weight—final egg weight)/initial egg weight] ×100; Values (means) within a column with uncommon letters are significantly (*p* < 0.05) different.

## References

[1] Estévez M. (2015). Oxidative damage to poultry: From farm to fork. Poult. Sci..

[2] Domínguez R., Pateiro M., Gagaoua M., Barba F.J., Zhang W., Lorenzo J.M. (2019). A comprehensive review on lipid oxidation in meat and meat products. Antioxidants.

[3] Surai P.F. (2002). Selenium in poultry nutrition 1. Antioxidant properties, deficiency and toxicity. World’s Poult. Sci. J..

[4] Pavlović Z., Miletić I., Jokić Ž., Pavlovski Z., Škrbić Z., Šobajić S. (2010). The effect of level and source of dietary selenium supplementation on eggshell quality. Biol. Trace Elem. Res..

[5] Gerhard N. (2000). Schrauzer Selenomethionine: A Review of Its Nutritional Significance, Metabolism and Toxicity. J. Nutr..

[6] Oliveira T.F.B., Rivera D.F.R., Mesquita F.R., Braga H., Ramos E.M., Bertechini A.G. (2014). Effect of different sources and levels of selenium on performance, meat quality, and tissue characteristics of broilers. J. Appl. Poult. Res..

[7] Utterback P.L., Parsons C.M., Yoon I., Butler J. (2005). Effect of supplementing selenium yeast in diets of laying hens on egg selenium content. Poult. Sci..

[8] Baylan M., Canogullari S., Ayasan T., Copur G. (2011). Effects of dietary selenium source, storage time, and temperature on the quality of quail eggs. Biol. Trace Elem. Res..

[9] Arpášová H., Petrovič V., Mellen M., Kačániová M., Čobanová K., Leng L. (2009). The effects of supplementing sodium selenite andselenized yeast to the diet for laying hens on thequality and mineral content of eggs. J. Anim. Feed Sci..

[10] Biswas A., Mohan J., Sastry K.V.H. (2006). Effect of higher levels of dietary selenium on production performance and immune responses in growing Japanese quail. Br. Poult. Sci..

[11] Abbaszadeh Mobaraki M., Aghdam Shahryar H. (2015). The Impact of Different Levels of Vitamin E and Selenium on the Performance, Quality and the Hatchability of Eggs from Breeding Japanese Quails. Iran. J. Appl. Anim. Sci..

[12] Ahmadian H., Nemati Z., Karimi A., Safari R. (2019). Effect of different dietary selenium sources and storage temperature on enhancing the shelf life of quail eggs. Anim. Prod. Res..

[13] Nemati Z., Alirezalu K., Besharati M., Amirdahri S., Franco D., Lorenzo J.M. (2020). Improving the quality characteristics and shelf life of meat and growth performance in goose fed diets supplemented with vitamin E. Foods.

[14] Łukaszewicz E., Korzeniowska M., Kowalczyk A., Bobak L. (2007). Effect of feed supplementation with organic selenium and vitamin e on chemical composition and sensory characteristics of japanese quail (coturnix japonica) eggs. Pol. J. Food Nutr. Sci..

[15] Kirunda D.F., Scheideler S.E., McKee S.R. (2001). The efficacy of vitamin E (DL-alpha-tocopheryl acetate) supplementation in hen diets to alleviate egg quality deterioration associated with high temperature exposure. Poult. Sci..

[16] Skřivan M., Marounek M., Dlouhá G., Ševčíková S. (2008). Dietary selenium increases vitamin E contents of egg yolk and chicken meat. Br. Poult. Sci..

[17] Surai P.F. (2002). Selenium in poultry nutrition 2. Reproduction, egg and meat quality and practical applications. World’s Poult. Sci. J..

[18] Mohiti-Asli M., Shariatmadari F., Lotfollahian H., Mazuji M.T. (2008). Effects of supplementing layer hen diets with selenium and vitamin E on egg quality, lipid oxidation and fatty acid composition during storage. Can. J. Anim. Sci..

[19] Urso U.R.A., Dahlke F., Maiorka A., Bueno I.J.M., Schneider A.F., Surek D., Rocha C. (2015). Vitamin E and selenium in broiler breeder diets: Effect on live performance, hatching process, and chick quality. Poult. Sci..

[20] Nutrient Requirements of Poultry 10th Revised Edition National Academies. https://www.nationalacademies.org/our-work/nutrient-requirements-of-poultry-10th-revised-edition.

[21] Skrivan M., Bubancova I., Marounek M., Dlouha G. (2010). Selenium and alpha-tocopherol content in eggs produced by hens that were fed diets supplemented with selenomethionine, sodium selenite and vitamin E. Czech J. Anim. Sci..

[22] Faustman C., Specht S.M., Malkus L.A., Kinsman D.M. (1992). Pigment oxidation in ground veal: Influence of lipid oxidation, iron and zinc. Meat Sci..

[23] Zhang W., Zhang K.Y., Ding X.M., Bai S.P., Hernandez J.M., Yao B., Zhu Q. (2011). Influence of canthaxanthin on broiler breeder reproduction, chick quality, and performance. Poult. Sci..

[24] Škrobánek P., Hrbatá M., Baranovská M., Juráni M. (2004). Growth of Japanese quail chicks in simulated weightlessness. Acta Vet. Brno.

[25] Amiri Andi M., Shivazad M., Pourbakhsh S.A., Afshar M., Rokni H., Shiri N.E., Mohammadi A., Salahi Z. (2006). Effects of vitamin E in broiler breeder diet on hatchability, egg quality and breeder and day old chick immunity. Pak. J. Biol. Sci..

[26] Shahriar H.A., Adl K.N., Nezhad Y.E., Nobar R.S.D., Ahmadzadeh A. (2008). Effect of dietary fat type and different levels of vitamin E on broiler breeder performance and vitamin E levels of egg. Asian J. Anim. Vet. Adv..

[27] Reis R.N., Vieira S.L., Nascimento P.C., Peña J.E., Barros R., Torres C.A. (2009). Selenium contents of eggs from broiler breeders supplemented with sodium selenite or zinc-L-selenium-methionine. J. Appl. Poult. Res..

[28] Abd-El-Maksoud A.A.A. (2006). Effect of vitamin E supplementation on performance of laying hens during summer months under the desert conditions. Egypt. Poult. Sci..

[29] Naylor A.J., Choctand M., Reinke N. (2004). Selenium supplementation affects broiler growth performance, meat yield and feather coverage. Br. Poult. Sci..

[30] Samli H.E., Okur A., Senkoylu N. (2005). Effects of Storage Time and Temperature on Egg Quality in Old Laying Hens. J. Appl. Poult. Res..

[31] Franco D., Rois D., Arias A., Justo J.R., Marti-Quijal F.J., Khubber S., Barba F.J., López-Pedrouso M., Lorenzo J.M. (2020). Effect of breed and diet type on the freshness and quality of the eggs: A comparison between MOS (Indigenous Galician breed) and Isa brown hens. Foods.

[32] Payne R.L., Lavergne T.K., Southern L.L. (2005). Effect of inorganic versus organic selenium on hen production and egg selenium concentration. Poult. Sci..

[33] Maysa B., El-Sheikh A., Abdalla E. (2009). The effect of organic selenium supplementation on productive and physiological performance in a local strain of chicken. 1—The effect of organic selenium (Sel-PlexTM) on productive, reproductive and physiological traits of Bandarah local strain. Egypt. Poult. Sci. J..

[34] Kucuk O., Sahin N., Sahin K., Gursu M.F., Gulcu F., Ozcelik M., Issi M. (2003). Egg production, egg quality, and lipid peroxidation status in laying hens maintained at a low ambient temperature (6 °C) and fed a vitamin C and vitamin E-supplemented diet. Vet. Med. (Praha).

[35] Paton N.D., Cantor A.H., Pescatore A.J., Ford M.J., Smith C.A. (2002). The effect of dietary selenium source and level on the uptake of selenium by developing chick embryos. Poult. Sci..

[36] Paton N.D., Cantor A.H., Pescatore A.J., Ford M.J. (2000). Effect of dietary selenium source and storage on internal quality and shell strength of eggs. Poult. Sci..

[37] Golubkina N.A., Papazyan T.T. (2006). Selenium distribution in eggs of avian species. Comp. Biochem. Physiol. B Biochem. Mol. Biol..

[38] Kralik G., Gajčević Z., Suchý P., Straková E., Hanžek D. (2009). Effects of dietary selenium source and storage on internal quality of eggs. Acta Vet. Brno.

[39] Sari M., Isik S., Onk K., Tilki M., Kırmizibayrak T. (2012). Effects of layer age and different plumage colors on external and internal egg quality characteristics in Japanese quails (Coturnix coturnix japonica)—European Poultry Science. Arch. Geflugelkd..

[40] Decuypere E., Tona K., Bruggeman V., Bamelis F. (2001). The day-old chick: A crucial hinge between breeders and broilers. World’s Poult. Sci. J..

[41] Lu C.-L., Baker R.C. (1987). Effect of pH and Food Ingredients on the Stability of Egg Yolk Phospholipids and the Metal-Chelator Antioxidant Activity of Phosvitin. J. Food Sci..

[42] Sinanoglou V.J., Strati I.F., Miniadis-Meimaroglou S. (2011). Lipid, fatty acid and carotenoid content of edible egg yolks from avian species: A comparative study. Food Chem..

[43] Bansal M.P., Kaur P. (2005). Selenium, a versatile trace element: Current research implications. Indian J. Exp. Biol..

[44] Soliman F.N., Rizk R.E., Brake J. (1994). Relationship between shell porosity, shell thickness, egg weight loss, and embryonic development in Japanese quail eggs. Poult. Sci..

[45] Ferit Gursu M., Sahin N., Kucuk O. (2003). Effects of vitamin E and selenium on thyroid status, adrenocorticotropin hormone, and blood serum metabolite and mineral concentrations of Japanese quails reared under heat stress (34 °C). J. Trace Elem. Exp. Med..

[46] Zduńczyk Z., Drazbo A., Jankowski J., Juşkiewicz J., Czech A., Antoszkiewicz Z. (2013). The effect of different dietary levels of vitamin e and selenium on antioxidant status and immunological markers in serum of laying hens. Pol. J. Vet. Sci..

[47] Sun X., Yue S., Qiao Y., Sun Z., Wang C., Li  H. (2020). Dietary supplementation with selenium-enriched earthworm powder improves antioxidative ability and immunity of laying hens. Poult. Sci..

